# Precipitation of Magnetic Iron Oxide Induced by *Sporosarcina pasteurii* Cells

**DOI:** 10.3390/microorganisms9020331

**Published:** 2021-02-07

**Authors:** Yang Wu, Guozheng Zhao, Hao Qi

**Affiliations:** 1 School of Chemical Engineering and Technology, Tianjin University, Tianjin 300072, China; yangw@tju.edu.cn (Y.W.); guozhenzhao@tju.edu.cn (G.Z.); 2 Key Laboratory of Systems Bioengineering of Ministry of Education, Tianjin University, Tianjin 300072, China

**Keywords:** microbial induced precipitation, *Sporosarcina pasteurii*, magnetic particles, urease

## Abstract

*Sporosarcina pasteurii* (*S. pasteurii*) is bacterium notable for its highly efficient urea degradation ability. Due to its high urease activity, *S. pasteurii* has been successfully utilized in applications including solidifying soil or sand, termed “bio-concrete”. In addition to calcium carbonate precipitation, urease isolated from the jack bean plant was recently demonstrated to induce the formation of magnetic iron oxide particles from soluble ferrous ion in a designed reaction. However, it remained unknown if a similar magnetic material could be formed using whole cells with high urease activity under biocompatible conditions. Here, we demonstrated that magnetic iron oxide with a highly ordered structure could be formed on the surface of *S. pasteurii* cells with a theoretical product of 1.17 mg in a 2-mL reaction. Moreover, the cells surrounded by the precipitated magnetic iron oxide maintained their viability. Due to the simple cultivation of *S. pasteurii*, the process developed in this study could be useful for the green synthesis of magnetic iron oxide, basic research on the mechanism of magnetic microbial-induced precipitation (MIP), and related engineering applications.

## 1. Introduction

Microbial-induced precipitation (MIP) is a common phenomenon caused by chemical changes in the solution due to microbial metabolism, resulting in the deposition of metal ions. Additionally, MIP is a promising technological tool that is ecofriendly and cost-effective [1,2]. MIP has been applied in many fields, including bio-bricks [3], self-healing concrete [4], ground improvement [5], and the bioremediation of metal ions [6]. The cells of many different microbial species can induce MIP by similar mechanisms. In general, these cells can increase the solution pH to make it alkaline and supply anions for precipitation. *Synechococcus* and sulfate-reducing bacteria are typical examples. *Synechococcus* can use CO_2_ to form HCO_3_^−^ in photosynthesis, thereby leaving free OH^−^ that makes the pH sufficiently alkaline for CaCO_3_ precipitation [7]. Sulfate-reducing bacteria can reduce HSO_4_^−^ or SO_4_^2−^ to S^2−^, increasing the concentration of S^2-^ for sulfide precipitation [2].

Recently, *Sporosarcina pasteurii* has become increasingly popular in MIP research due to its ultra-high urease activity, which is, respectively, 100- and 14-fold higher than that of soybean urease and sword bean urease. Consequently, *S. pasteurii* cells can efficiently hydrolyze urea to generate NH_4_^−^, OH^−^, and HCO_3_^−^, leading to an increased pH and enough anions for the precipitation of metal ions. However, most studies focus on calcium carbonate precipitation induced by *S. pasteurii*, with limited research on other metal ions. Nevertheless, it is feasible for hydroxide and carbonate released by *S. pasteurii* to precipitate other metal ions.

(1) $${\left({\mathrm{NH}}_{2}\right)}_{2}\mathrm{CO}+3H_{2}O\overset{\mathrm{urease}}{\to }2{\mathrm{NH}}_{4}^{+}+{\mathrm{OH}}^{-}+{\mathrm{HCO}}_{3}^{-}$$

(2) $${\mathrm{Fe}}^{2+}+2{\mathrm{OH}}^{-}=\mathrm{Fe}{\left(\mathrm{OH}\right)}_{2}$$

(3) $$6\mathrm{Fe}{\left(\mathrm{OH}\right)}_{2}+O_{2}=2{\mathrm{Fe}}_{3}O_{4}+6H_{2}O$$

Magnetic iron oxide particles are important materials in research and engineering due to their permanent magnetism. Magnetic particles have been applied in different areas, such as wastewater purification [8], electrode materials for supercapacitors [9], biomedicine [10], and protein purification using magnetic beads [11]. However, the traditional preparation methods require high temperature, high pressure, and hazardous solvents. Some biotic routes of iron-containing compounds have been reported, including *Actinobacter* spp. [12,13,14] and *Rhodococcus erythropolis* [15,16]. If magnetic particles could be produced by MIP, this would offer many advantages over traditional methods. Interestingly, the synthesis of magnetic iron oxide particles using urease has been reported [17]. After urea is hydrolyzed by urease (Equation (1)), the pH increases due to the release of hydroxyl ions, and ferrous hydroxide is produced (Equation (2)). When oxygen enters the solution, ferrous hydroxide is oxidized into ferroferric oxide (Equation (3)). At different reaction temperatures, magnetic particles of various morphologies can be obtained, including nanospheres, nanosheets, and nanorods. Since the key catalyst for this method is urease, we concluded that it should also be possible to synthesize magnetic particles using *S. pasteurii*, which acts as a whole-cell urease catalyst for particle production.

In this study, a magnetic iron oxide precipitate was firstly formed via MIP induced by the whole cells of *S. pasteurii*. The reaction conditions were successfully optimized to allow the use of a simple solution at mild temperatures. Furthermore, the microstructure of the resulting particles was investigated, revealing a highly ordered sheet structure on the cell surface. Finally, it was found that *S. pasteurii* cells covered by the magnetic material remained viable, making possible a wider range of MIP applications in the future. To the best of our knowledge, this is the first study to synthesize magnetic iron oxide using whole cells of *S. pasteurii* by MIP.

## 2. Materials and Methods

### 2.1. Strain and Culture Conditions

*Sporosarcina pasteurii* (ATCC 11859) was used in the MIP experiment in this study. The bacterium was cultured in YE-urea medium (20-mg/mL yeast extract, 10-mg/mL (NH_4_)_2_SO_4_, 15.75-mg/mL Tris, and 20-mg/mL urea) at 30 °C and 220 rpm. YE-urea plates were produced by adding 15-mg/mL agar. Before inoculation, all components except the urea solution were autoclaved at 121 °C for 20 min, while the urea solution was filtered through a 0.22-µm pore size membrane.

To determine the growth curves, the optical density at 600 nm (OD_600_) of a 100-mL *S. pasteurii* culture was measured every two hours. A single colony of *S. pasteurii* was picked from a YE-urea plate and used to inoculate 5 mL of YE-urea medium for overnight culture at 30 °C and 220 rpm. Then, 1 mL of the resulting overnight culture was used to inoculate 100 mL of fresh YE-urea medium in a 250-mL shake flask and cultured at 30 °C and 220 rpm. The OD_600_ of the flask culture was measured every two hours.

### 2.2. Magnetic MIP Induced by S. pasteurii Cells

Magnetic MIP was carried out by suspending *S. pasteurii* cells from an actively growing culture in FeCl_2_-urea solution (400-mM urea and 8-mM FeCl_2_). The bacterial suspension was prepared from a culture with OD_600_ of 2.0, which was washed three times with the same volume of Buffer A (100-mM NaCl, 10-mM KCl, and 20-mM MgCl_2_, pH7.0). Then, the bacterial pellet was resuspended in an equal volume of Buffer A for the magnetic MIP reaction. To prepare the FeCl_2_-urea solution, ultrapure water was boiled and cooled to room temperature to remove oxygen. Urea and FeCl_2_ were added to the cooled water to a final concentration of 400 mM and 8 mM, respectively. Finally, the bacterial suspension and FeCl_2_-urea solution were combined in a centrifuge tube at a ratio of 1:1 and reacted at 30 °C and 220 rpm for 30 min. The concentrations of some ingredients were changed according to the research designs described in the results section.

### 2.3. Determination of the FeCl_2_ Concentration Using the 5-Sulfosalicylic Acid (SSA) Method

The concentration of FeCl_2_ was determined using the SSA method [18], with minor modifications, as follows. Firstly, 25% aqueous ammonia was added to the test solution to oxidize divalent Fe^2+^ into Fe^3+^ ions and increase the pH. Secondly, 10% (*w*/*v*) SSA solution was added to form a yellow complex with Fe^3+^ ions. The yellow complex exhibited maximal absorbance at 425 nm and was stable for more than 48 h. Next, the absorbance of FeCl_2_ solutions with known concentrations was measured to construct the standard curve. Finally, the same assay was performed with samples of unknown concentrations.

### 2.4. Optical Microscopy and Scanning Electron Microscope (SEM) Analysis

Optical microscopy observation of MIP was carried using a DMi8 inverted microscope (Leica, Wetzlar, Germany), equipped with an EMCCD camera (Andor, Belfast, UK) and an Adaptive Focus Control system. When the magnetic MIP reaction was finished, the final product was collected by centrifugation and washed three times with ultrapure water. The sample was resuspended in ultrapure water and spread on the glass slide for microscopy.

SEM and energy-dispersive spectroscopy (EDS) analysis were carried out on a S-4800 SEM (Hitachi, Japan) equipped with an EDS device. To prepare the SEM samples, the precipitation product was collected by centrifugation (7500× *g*, 2 mL, 5 min), washed three times with ultrapure water, resuspended in ultrapure water, and spread on the cover glass, which was thin enough for SEM. The cover glass with attached sample was air-dried thoroughly and coated with an ultrathin conductive gold film before SEM observation.

### 2.5. Microbial Activity Assay after MIP

After the MIP reaction, the pellet was collected by centrifugation and washed three times with Buffer A at pH 7.0, 5.0, and 3.0, which was adjusted with HCl. Then, the samples were resuspended in Buffer A, spread on YE-urea plates, and incubated at 30 °C for 36 h. The numbers of colonies on these plates were counted to calculate the viability.

## 3. Results

### 3.1. Magnetic MIP Induced by Cells of S. pasteurii

**Figure 1:**
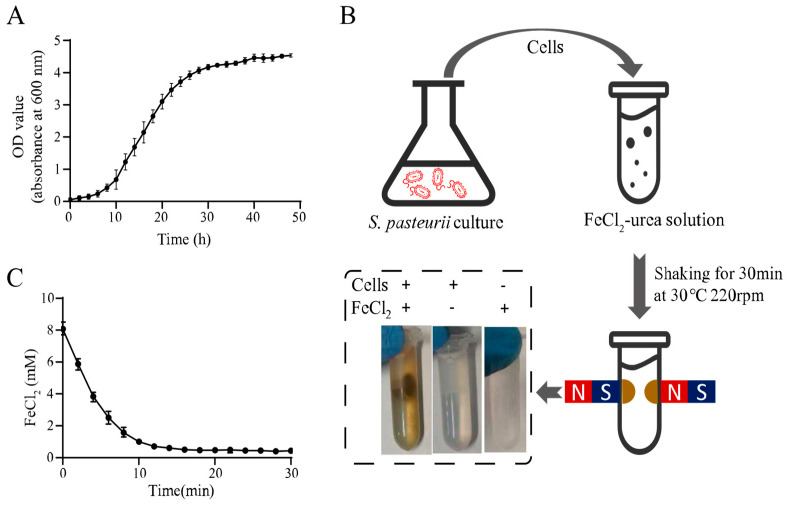
Magnetic microbial-induced precipitation (MIP) induced by whole cells of *Sporosarcina pasteurii*. (**A**) Growth curve of *S. pasteurii.* (**B**) Schematic diagram of magnetic MIP induced by *S. pasteurii*. (**C**) Curve of residual FeCl_2_ in the supernatant during magnetic MIP. All values were determined in three independent biological replicates.

The urease activity of *S. pasteurii* plays an important role in MIP, and it is highest in the exponential growth phase [19]. Thus, *S. pasteurii* cells in the exponential phase were utilized in this study. According to the *S. pasteurii* growth curve shown in Figure 1A, the culture reached the exponential phase at an OD_600_ of 2.0, and the corresponding cells were used for the magnet mineralization experiment.

The harvested cells were mixed with the FeCl_2_-urea solution and shaken at 30 °C for 30 min. Notably, when a magnetic field was applied to the reaction tube, the precipitate produced by magnetic MIP was concentrated around the magnets. This was a significant result demonstrating the magnetism of the MIP product. In contrast with this result, no magnetic pellet was found in the absence of cells or FeCl_2_, which indicated that both cells and FeCl_2_ were essential for magnetic MIP product formation. To unequivocally verify the urease activity involved in iron oxide formation, the uninduced bacterial cells, which were inoculated in the YE and LB mediums without urea, were tested in the same MIP reaction. A similar magnetic MIP product was also witnessed in Figure S1.

In order to confirm that FeCl_2_ takes part in magnetic MIP and monitor the reaction progress, the FeCl_2_ concentration in the supernatant was measured every two min during the MIP reaction using the SSA method described before. Figure 1C shows the time profile of the FeCl_2_ concentration, showing that it decreased rapidly from the reaction start, after which, the rate of decrease slowed over time. Finally, the lowest concentration was reached at about 14 min. This result indicated that FeCl_2_ was involved in magnetic MIP, and the reaction reached an equilibrium in 14 min. The final FeCl_2_ concentration in the supernatant was 0.43 ± 0.08 mM in 30 min. This meant that 7.57 ± 0.08-mM FeCl_2_ was precipitated from the supernatant, which showed 94.63% ± 1.00% efficiency of transformation, theoretically. With a 2-mL volume of each test, about 1.17 mg of product could be produced in one reaction. For the production of 1-g precipitate, a 1709.40-mL volume of reaction should be performed, which required 1.73-g FeCl_2_, 75.26-g urea, 34.19-g yeast extract, 17.09-g (NH_4_)_2_SO_4_, and 26.92-g Tris.

### 3.2. Characterization of the Magnetic MIP

**Figure 2:**
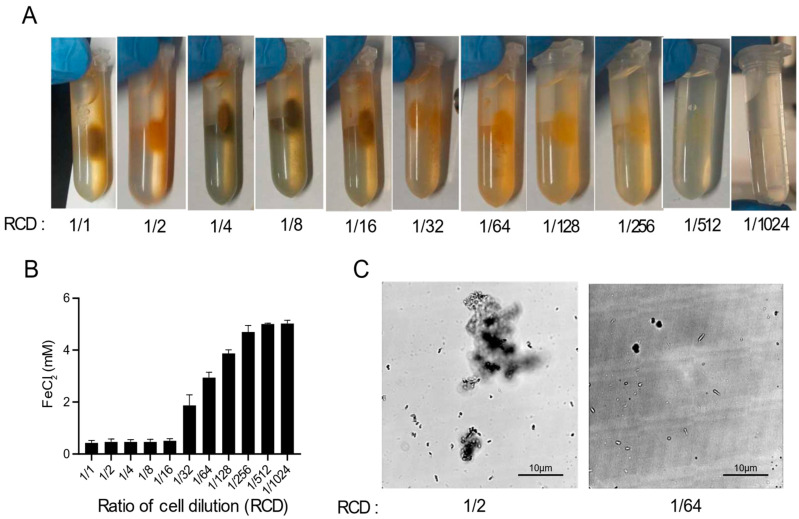
Optimization of magnetic MIP. (**A**) Photos of magnetic MIP with different ratios of cell dilutions (RCDs). (**B**) Residual FeCl_2_ concentrations in the supernatant of magnetic MIP with different RCDs were measured in three biological replicates. (**C**) Microscopic observation of the morphology of precipitates in magnetic MIP with RCDs of 1/2 and 1/64.

As shown in Figure 1B, there was no magnetic pellet in the absence of cells. To study the effects of cells in magnetic MIP, a series of experiments with different concentrations of cells were designed. Cell suspensions with eleven different concentrations were made by two-fold serial dilution in Buffer A, and the final concentrations ranged from 1/1 to 1/1024 of the original cell suspension (initial OD_600_ = 2.0). Then, these different cell suspensions were mixed with the FeCl_2_-urea solution to induce a magnetic MIP reaction. As shown in Figure 2A, the magnetic precipitation reduced with the decrease of the cell concentrations. Hence, there was a clear trend in which the cell concentration and precipitation were positively correlated.

In order to quantify the MIP at different cell concentrations, the FeCl_2_ concentrations in the supernatant were measured suing the 5-sulfosalicylic acid (SSA) method (Figure 2B). The FeCl_2_ in the reaction mixture could either precipitate or remain in the supernatant, and the remaining iron ions, after subtracting the concentration in the supernatant, can be considered to form the precipitate. Thus, the MIP reaction progress can be quantitatively monitored by the determination of the residual iron ions in the supernatant. As shown in Figure 2B, the residual FeCl_2_ concentration increased with the decrease of the cell concentration, which was generally consistent with the results shown in Figure 2A. However, the residual FeCl_2_ concentration and the cell concentration were only moderately negatively correlated when the cell concentration ranged from 1/16 to 1/256. At cell concentrations ranging from 1/1 to 1/8, the residual FeCl_2_ concentration was almost zero, which suggested that the excess amount of cells released too much OH^−^ and HCO_3_^−^ in the reaction, resulting in almost all the FeCl_2_ forming the precipitate. Conversely, when the amount of cells was too small, ranging from 1/512 to 1/1024, almost no FeCl_2_ was converted into the precipitate.

The morphology of the precipitates formed at different cell concentrations was also observed using optical microscopy (Figure 2C). In the 1/2 cell concentration reaction, the precipitate was agglomerated in a cluster form, which suggested that enough iron oxide was precipitated to aggregate the cells into a mass. By contrast, the precipitate presented as single cells at the 1/64 cell concentration due to insufficient iron oxide precipitation. At all cell concentrations, the cells remained stationary under the microscope, showing no activity or swimming movement.

**Figure 3:**
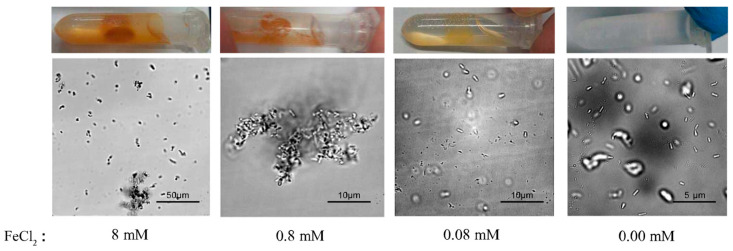
Effect of FeCl_2_ concentrations on magnetic MIP. Macroscopic photos and microscopic morphology of precipitates from magnetic MIP with 8-mM, 0.8-mM, 0.08-mM, and 0.00-mM FeCl_2_ are shown.

Next, we assessed the effects of FeCl_2_ concentration, which is also essential for the magnetic MIP reaction. Accordingly, a series of magnetic MIP experiments with FeCl_2_-urea solutions containing 8-mM, 0.8-mM, 0.08-mM, or 0.00-mM FeCl_2_ were prepared. Then, these FeCl_2_-urea solutions were mixed with the cell suspension and shaken at 30 °C for 30 min. As shown in Figure 3, the magnetic precipitation decreased with the decrease of FeCl_2_ concentration. No precipitation was observed in the reaction with 0.00-mM FeCl_2_, as expected. Figure 3 shows the microscopic morphology of the precipitates formed at different FeCl_2_ concentrations. In the reaction with 8-mM FeCl_2_, the precipitate was mostly agglomerated, with few single cells. Under real-time observation of the microscope, microbial swim could be witnessed, which was regarded as a parameter of cell activity. No traces of microbial movement were observed in the 8-mM FeCl_2_ reaction, implying inactive cells. In the reaction with 0.8-mM FeCl_2_, the precipitate presented large agglomerations without any single cells. Different from no traces of microbial movement in the 8-mM reaction, the large agglomerates would suddenly rotate or move in some cases, implying that some cells were active. In the reaction with 0.08-mM FeCl_2_, no agglomerated precipitate was found. All single-cell precipitates rotated or moved slowly in situ, implying that all cells were active. By contrast, no precipitation was observed in the 0.00-mM FeCl_2_ reaction, in which the cells remained highly active, with quick swimming movements.

**Figure 4:**
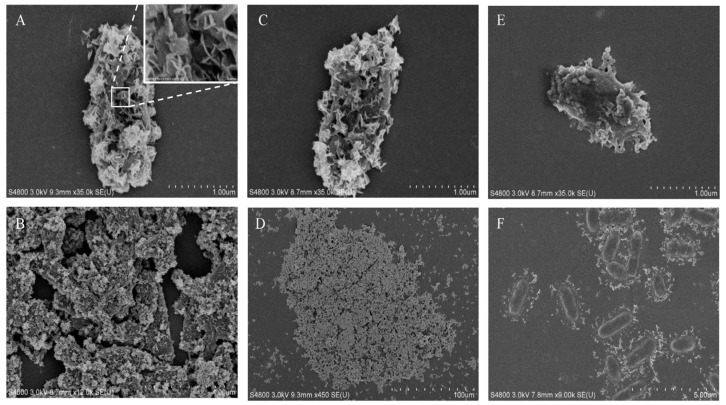
Scanning electron microscope (SEM) analysis of precipitates obtained in magnetic MIP. SEM analysis was applied on precipitates from magnetic MIP with 8-mM (**A**,**B**), 0.8-mM (**C**,**D**), 0.16-mM (**E**), and 0.08-mM (**F**) FeCl_2_.

To better capture the microscopic morphology of the magnetic precipitates, SEM was used for high-resolution observations (Figure 4). The single-cell precipitates are shown in Figure 4A, in which the cell surface was thoroughly covered with a serrated sheet of precipitate. This was very different from SEM images of microbiological-induced calcium carbonate precipitation (MICP) shown in Figure S2. MICP presented spherical precipitates with void traces of cells, implying that the cells were washed out after inducing calcium carbonate precipitation. Cells were also visible around the MICP precipitates shown in Figure S1B,D. By contrast, the cells remained in the magnetic MIP precipitate. Notably, a previous study reported magnetic Fe_3_O_4_ nanosheets formed using urease at 30 °C [19], which was consistent with the serrated sheet morphology of precipitate in this study. The previous study also prepared Fe_3_O_4_ nanospheres and nanorods at different temperatures, indicating that it might be possible to produce MIP of similar morphology using *S. pasteurii* in future studies. As can be seen in the SEM images of precipitates from the reactions with 8- and 0.8-mM FeCl_2_ (Figure 4A–D), the cells were completely covered with magnetic nanosheets and gathered into large groups. When the FeCl_2_ concentration was decreased to 0.16 mM (Figure 4E), the nanosheets only covered a part of the microbial surface. However, there was a significant difference in the SEM images of precipitates from the 0.08-mM FeCl_2_ (Figure 4F). The precipitate was scattered around the cells instead of sticking to the cell surface. Here, a hypothesis was suggested that the initial site of MIP might be the pili or flagella around the cells. Then, the precipitate gradually spread from the pili to the surface of the cells, partially covering the microbial surface (Figure 4E). As the reaction progressed further, the cells were completely covered with nanosheets and finally gathered into large groups. However, this hypothesis still needs more evidence to be proven in the future. Finally, energy-dispersive spectroscopy (EDS) was also applied on the samples shown in Figure 4A,E. The results shown in Figure S3 demonstrate that iron and oxygen were present in the nanosheets on the cell surface. Since the detection depth of EDS was 1 µm, the elements present inside the microbial cells under the nanosheets were also included in the EDS results, which led to the abnormally high ratio of oxygen. However, the magnetic properties of the iron oxide particles were shown in the magnetic adsorption and EDS detection. The particles were concentrated around the magnets in the magnetic field. The magnetic nanosheets containing iron were first observed on the cell surface.

### 3.3. Viability of Cells Covered with the Magnetic Precipitates

**Figure 5:**
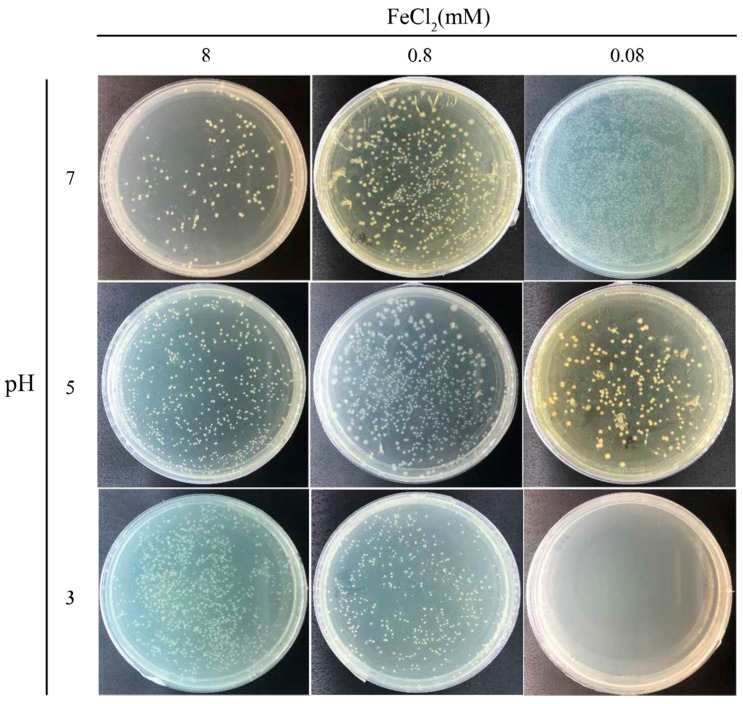
Cell viability after magnetic MIP. Photos of plates of MIP cells treated using buffers of different pH to release cells from the precipitates.

After MIP, the cells were covered in the magnetic precipitate, and it was interesting to investigate whether the engulfed cells were still active. However, the magnetic precipitate covering the cells made it difficult to distinguish if they were dead or alive. To dissolve the cover, the magnetic precipitate was resuspended in Buffer A with pH levels of 7.0, 5.0, and 3.0 and spread on YE-urea plates to culture the viable cells. The number of colonies on these plates (Figure 5) were quantified to calculate the viable count. In the 8-mM FeCl_2_ reaction, the number of colonies was increased with the decreasing pH level of Buffer A. Most magnetic particles were precipitated on the cell surface in this case. With the pH decrease of Buffer A, more magnetic precipitates were dissolved, resulting in more cells being released from the aggregates to form individual colonies. In the 0.8-mM FeCl_2_ reaction, most colonies were observed in the experiment with Buffer A (pH 5.0). In this case, an intermediate amount of magnetic particles was precipitated on the cell surface, which may have helped the cell resist the weak acid. However, the cells were killed after complete dissolution of the magnetic precipitate in the strongly acid solution. In the 0.08-mM FeCl_2_ reaction, the number of colonies decreased with the decreasing pH level of Buffer A. Without complete protection by the magnetic precipitate, the cells were easily killed by the acid solution. Additionally, without the magnetic MIP reaction, the cell viability of *S. pasteurii* was also tested in acid buffer treatment. Cells were completely killed by the acid damage, leading to no colony in the plate (Figure S4). Overall, these results indicate that the cells trapped in the magnetic precipitate were active, and the magnetic precipitate could help shield the cells from acid damage.

## 4. Discussion

Magnetic iron oxide precipitates induced by *S. pasteurii* cells were first obtained in this study, providing a relatively promising method for magnetic iron oxide production. The biocompatible precipitation conditions were successfully optimized to allow the use of a simple solution at a mild temperature. The resulting method for preparing magnetic iron oxide particles is therefore greener than the traditional methods, which require large amounts of energy and hazardous solvents. Since the reaction was catalyzed by whole cells of *S. pasteurii* instead of the purified urease that was employed in previous studies, the method was both faster and less expensive. With such attributes, this whole-cell biocatalytic method can be applicated in various fields with great potential.

Another important finding was that the magnetic precipitate exhibited a highly ordered sheet structure on the surface of *S. pasteurii* cells. The ordered structure may be due to consistent chemical crystallization. Precipitation on *S. pasteurii* can be used as a model of bacterial cell surfaces as nucleation sites in MIP. Due to the magnetic material on the surface, the cells could gather into large groups by adhesive forces or magnetic forces, and the large magnetic aggregates were more conducive to separation in a magnetic field. By contrast, CaCO_3_ precipitation in MICP produced spherical particles outside the cells. The nucleation of MICP may start on the surface of *S. pasteurii*, but the cell can escape after inducing CaCO_3_ precipitation, leaving voids traces in the surface of the CaCO_3_ particles. Accordingly, numerous free cells were observed surrounding the precipitated particles. The different morphology of the precipitates suggested different dynamic crystallization mechanisms.

The results shown in Figure 4 may support the hypothesis that the initial site of nucleation of dynamic precipitation was the pili or flagella of *S. pasteurii*. As can be seen in Figure 4F, the precipitate was scattered around the cells, instead of sticking to the cell surface, which may represent the initial form of the crystals. Moreover, *S. pasteurii* surrounded by the magnetic material kept its cell activity, which indicated that the formation of the magnetic material did not affect the intracellular components. Accordingly, the *S. pasteurii* in the magnetic material can potentially be reused. These results provide insights into the nucleation dynamics of MIP, providing a basis for further research.

However, there were still some considerations in the present study. Firstly, in need of the scale-up production of 100-g magnetic particles, a 170.94-L volume of reaction was performed, which required 173-g FeCl_2_, 7526-g urea, 3419-g yeast extract, 1709-g (NH_4_)_2_SO_4_, and 2692-g Tris. The liquid waste should be sterilized for biosafety and disposed harmlessly, containing microbial cells, nutrients, and the residual FeCl_2_-urea solution. Secondly, microbial cells were packaged in magnetic iron oxide particles, which may lead to complicated biological contamination in some applications. However, it also provided the possibility of highly active urease cell separation through a magnetic force and cell recycling in pollution bioremediation. Finally, the sizes of the particles varied from 1 µm to 100 µm, according to the SEM results, which need optimization for the uniformity of particles. Looking forward, the whole-cell catalyst established in this study has several practical applications. Firstly, it allows the possibility of the green, simple, and inexpensive preparation of magnetic particles. Secondly, it was observed that the addition of Fe_3_O_4_ particles can increase the soil strength in MICP [20]. Thus, mixed precipitations of ions such as Ca^2+^ and Fe^2+^ are likely to improve the soil strength. Finally, cells in the magnetic precipitates were active and can likely be reused in many applications, such as sewage treatment and the bioremediation of heavy metal pollution.

## 5. Conclusions

The present study clearly demonstrates an ecofriendly green approach for the microbially induced precipitation of magnetic iron oxide particles using *S. pasteurii* with a theoretical product of 1.17 mg in a 2-mL reaction. To investigate the mechanism of precipitation, SEM was applied to observe the precipitate morphology, which displayed a highly ordered sheet structure on the cell surface. The highly efficient urease produced by *S. pasteurii* plays a key role in the formation of ordered magnetic materials, and the cells remained viable inside the precipitates. The results of this study could be useful for some ecofriendly engineering applications and mechanism research of magnetic MIP.

## Data Availability

The data presented in this study are available on request from the corresponding author.

## Supplementary Materials

The following materials are available online at https://www.mdpi.com/2076-2607/9/2/331/s1: Figure S1: Magnetic MIP reaction with uninduced bacterial cells, Figure S2: SEM analysis of *S. pasteurii*-induced calcium carbonate precipitation (MICP), Figure S3: Energy-dispersive spectroscopy (EDS) analysis of precipitates from magnetic MIP, and Figure S4: Cell viability of *S. pasteurii* treated by acid buffer without magnetic MIP reaction.
