# Supplementary material for: Precipitation of Magnetic Iron Oxide Induced by Sporosarcina pasteurii Cells
Source: Microorganisms. 2021 Feb 7;9(2):331. doi: 10.3390/microorganisms9020331 (PMC7916055; doi:10.3390/microorganisms9020331)
Supplement: Supplementary file 1 [file microorganisms-09-00331-s001.pdf]

Supplementary Materials

Precipitation of magnetic iron oxide induced by *Sporosarcina pasteurii* cells

Yang Wu [1,2], Guozheng Zhao[1,2] and Hao Qi[1,2,*]

[1] School of Chemical Engineering and Technology, Tianjin University, Tianjin, China

[2] Key Laboratory of Systems Bioengineering of Ministry of Education, Tianjin University, Tianjin, China

Correspondence should be addressed to Hao Qi (haoq@tju.edu.cn)

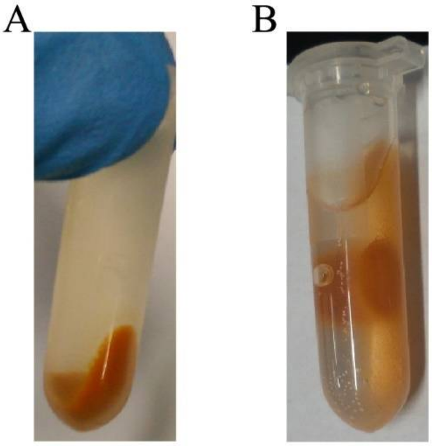

Figure S1. Magnetic MIP reaction with uninduced bacterial cells. Similar magnetic MIP products were witnessed using cells cultured in the YE medium without urea (A) and LB medium without urea (B).

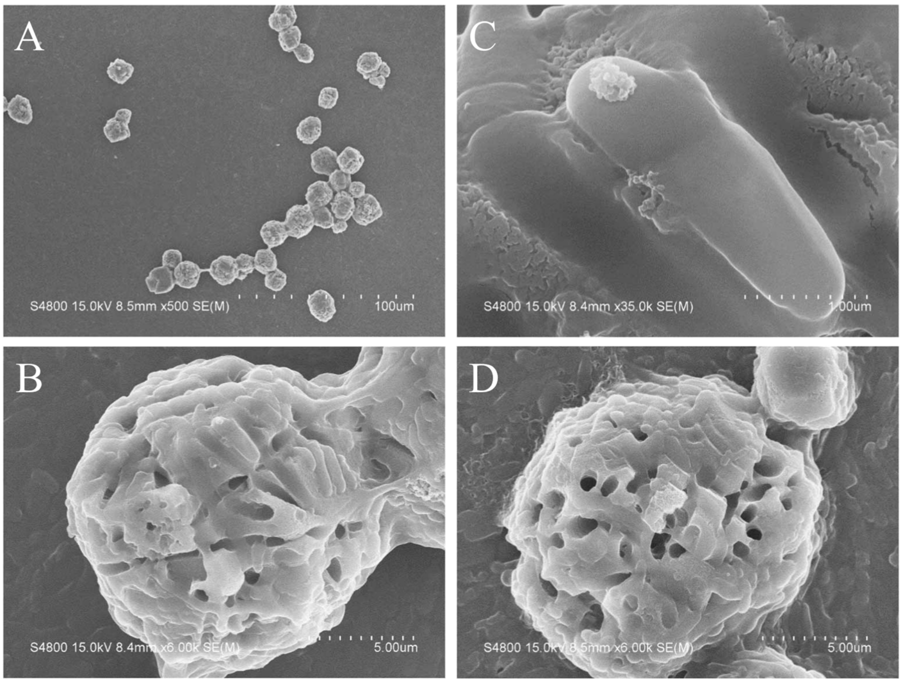

Figure S2. SEM analysis of *S. pasteurii* induced calcium carbonate precipitation (MICP).

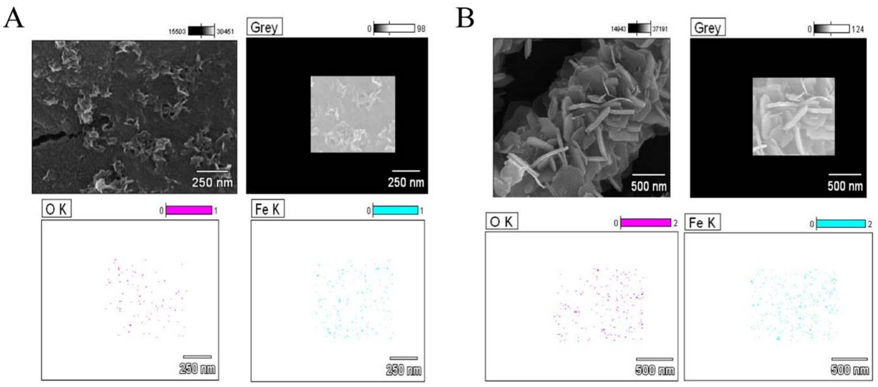

A

	Net Counts	Wt.%	Atom %
O K	19	46.52	75.23
Fe L	0	---	---
Fe K	9	53.48	24.77
Total		100	100

B

	Net Counts	Wt.%	Atom %
O K	24	36.15	66.40
Fe L	5	---	---
Fe K	19	63.85	33.60
Total		100	100

Figure S3. Energy dispersive spectroscopy (EDS) analysis of precipitates from magnetic MIP. Precipitates from magnetic MIP with 8 mM (A) and 0.16 mM (E) $FeCl_2$ were analyzed by EDS.

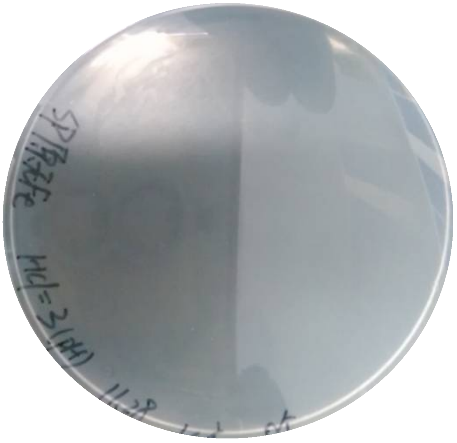

Figure S4. Cell viability of *S. pasteurii* treated by acid buffer without magnetic MIP reaction. Without iron oxide shielding, cells were completely killed by acid damage, leading to no colony in the plate.
